# Development of an AI model to predict tooth movement during orthodontic treatment

**DOI:** 10.6026/973206300220675

**Published:** 2026-02-28

**Authors:** Ushanandhini K, Ashish Kumar, Abhita Malhotra, Kavyashree G, Esther Lhingneihoi Mate, Shobhit Saxena

**Affiliations:** 1 Department of Orthodontics & Dentofacial Orthopedics, Sri Ramakrishna Dental College & Hospital, Coimbatore, India; 2 Department of Orthodontics and Dentofacial Orthopaedics, NIMS Dental College & Hospital, NIMS University, Rajasthan, Jaipur, India; 3 Department of Orthodontics and Dentofacial Orthopaedics, Manav Rachna Dental College, Faridabad, India; 4 Department of Periodontology, The Oxford Dental College, Bengaluru, Karnataka, India; 5 Department of Prosthodontics, Kalinga Institute of Dental Sciences, KIIT Deemed To Be University, Bhubaneswar, Odisha, India; 6 Department of Orthodontics and Dentofacial Orthopaedics, Narsinhbhai Patel Dental College and Hospital, Sankalchand Patel University, Visnagar, India

**Keywords:** Artificial Intelligence, orthodontic tooth movement, deep learning, treatment prediction, digital orthodontics

## Abstract

Accurate prediction of three-dimensional tooth movement remains a major challenge in orthodontic treatment planning, with conventional methods showing error rates of 30-50% for complex movements. Therefore, it is of interest to develop and evaluate an artificial intelligence model for predicting orthodontic tooth movement using digital treatment records and intraoral scan data. A deep learning framework combining convolutional neural networks and recurrent neural networks was trained on 4,218 orthodontic cases comprising 892,476 individual tooth movement records across multiple treatment stages. The model achieved an overall prediction accuracy of 91.3%, with a mean absolute error of 0.24 mm for linear movement and 1.87°C for angular movement, significantly outperforming traditional prediction approaches (p = 0.001). Thus, we show that AI-based tooth movement prediction can enhance orthodontic treatment planning accuracy, reduce chairside time and improve overall clinical outcomes.

## Background:

The core of orthodontic treatment planning is the possibility to predict the response of the teeth to the forces applied to them during the years of the treatment and the proper prediction of tooth movement is still regarded as one of the most difficult issues of clinical orthodontics [1]. Biological response to orthodontic forces-When orthodontic forces are present there are intricate interactions between the mechanical stimuli, periodontal ligament remodelling, bone metabolism and patient- specific factors which are hard to measure and predict according to the traditional approaches [2]. Although the orthodontic biomechanics and orthodontic treatment technologies have improved considerably, there has still been a tendency of prediction errors on the intended and the actual tooth positions that influence the efficiency of treatment and its outcomes [3]. The use of digital treatment planning systems that create virtual environments representing the expected end-outcome tooth positions and staging is becoming more and more common to orthodontic practice [4]. Clear aligner systems, specifically, require movement predictions to great precision in order to produce sequential aligners which gradually move the teeth to desired positions [5]. Research has however shown that there is a significant variation in the accuracy of the projected movements resulting in some movement types having less than half the projected displacement [6]. Such differences in prediction require in-course corrections, more aligners and more time of treatment which affects clinical effectiveness as well as patient satisfaction [7]. The factors affecting the accuracy of prediction of orthodontic tooth movement are multifactorial and they involve the type of movement, the magnitude of movement, tooth structure, root structure, bone density, periodontal condition, age of patients and compliance variables [8].

The traditional biomechanical models seek to model these variables through finite element analysis and mathematical models but the biological variation in nature of the patients constrains the accuracy of physics-based predictions [9]. It has been found that there can be a two to fivefold difference between individual patient responses to the same force systems necessitating the constraints of a single-size-fits all prediction algorithms [10]. In particular, technologies based on artificial intelligence and machine learning have proven to have exceptional results in detecting complex patterns within large amounts of data and also making predictions that take into consideration a variety of interacting variables at the same time [11]. In medicine, deep learning models have already demonstrated the capabilities of the top medical professionals in the field of diagnostic imaging, treatment outcome prediction and customized medicine in a wide variety of fields [12]. The usage of AI in dentistry has grown fast and those solutions include caries screening, periodontal evaluation, cephalometric analysis and treatment planning support [13]. Recent studies have been initiated into the use of AI specifically in orthodontic therapy, such as automated recognition of cephalometric landmarks, malocclusion detection and treatment requirement, as well as extraction advisor [14]. A number of researchers have shown that it is possible to use machine learning to forecast the results of treatment and its duration using initial documentation [15]. Nevertheless, there is a relative lack of research on the use of AI to predict the movements of individual teeth in the stages of sequential treatment [16]. Modern orthodontic practices have created a lot of digital data offering unprecedented possibilities to train the AI model based on real treatment outcomes [17].

The intraoral scanning technology creates accurate three-dimensional records at various treatment times, allowing measurement of the movements of teeth that have been achieved that can be compared to the planned movements [18]. The combination of this richness of outcome data with pre-treatment features and exerted forces forms the basis of the development of predictive models that learn the truth of real clinical experience [19]. The abilities of deep learning structures to automatically locate the relevant features of the complex spatial data and detect the temporal patterns within the sequence of treatment phases make them particularly relevant to orthodontic movement prediction [20]. Convolutional neural networks are better at handling geometric data of 3D form, whereas recurrent neural networks are able to handle the dynamics of biological tooth movement response with time [21]. A combination of these architectures can possibly provide more predictive accuracy than conventional biomechanical methods. Although AI has a promising potential in orthodontics movement prediction, there are still serious gaps in the literature on how models should be developed, validated and applied to clinical practice [22]. The available literature has used fairly small databases and has been investigating individual movement modes or appliance systems and no extensive validation with traditional prediction techniques [23]. Moreover, the fact that AI predictions can be interpreted and integrated into a clinical workflow is to be considered with great care to be successfully implemented [24]. Therefore, it is of interest to design and test a powerful artificial intelligence model to predict tooth movement in 3D during orthodontic therapy using a deep learning model that was trained on a massive dataset of digital treatment cases. Secondary objectives were comparison to conventional prediction methods, analysis of movement specific accuracy and the analysis of factors that affected prediction performance.

## Materials and Methods:

## Study design and data source:

This was a retrospective cohort study based on digital orthodontic records of a multi-center data repository of the treatment data of 23 orthodontic practices based in North America and Europe and collected between January 2018 and December 2023. The coordinating center provided institutional review board approval and agreements on data sharing were made with the participating practices. Data were de-identified before the analysis in accordance with the data protection rules.

## Sample selection:

The screening of treatment records was done based on established eligibility criteria. Inclusion criteria included: completed orthodontic treatment with reported final outcomes; presence of pre-treatment intraoral scans and records of treatment planning; sequential intraoral scans at strong three treatment time points; complete permanent dentition between second premolar and second premolar; and treatment with either clear aligners or faceted appliances with digital planning. Inclusion criteria were: no case of surgery orthodontics; craniofacial syndrome or cleft lip/palate; severe periodontal disease with tooth mobility; incomplete records or no scan data; use of temporary anchorage devices; and prematurely terminated treatment. After screening, 4218 cases were found to qualify with 2,847 cases of clear aligners (67.5) and 1,371 cases of fixed appliances (32.5). The number of records of a single case had 892,476 records of individual tooth movement at successive treatment stages.

## Data processing and extraction:

The intraoral scans were converted to STL format and standard tooth position parameters were extracted through the custom Python scripts. Tooth centroid positions were used as the representation of the tooth (x, y, z position), angulation (mesio-distal tip) and inclination (labio-lingual torque) and rotation (around the long axis). The consecutive treatment stages movement vectors were determined on each tooth.

The pre-treatment variables were summarized as follows: demographics of the patient (age, sex); type of malocclusion; crowding/spacing; overbite and overjet; size of individual teeth; estimation of the root length based on panoramic radiograph; type of periodontal biotype.

## Planning data with treatment:

The planned movement vectors in each stage; force system specifications (where available); habits of the applier cases of attachers; and wire sequences of appliance cases with fixed appliances. Achieved vectors of movement were the outcome data and were derived based on sequential intraoral scans, which formed the ground truth in training and validation of the model.

## Pre-processing and augmentation of data:

Pre-processing of raw scan data involved: standardization of coordinate systems by occlusal plane alignment; scaling of the scanners to eliminate variations between scanners; identification and elimination of outliers due to biologically implausible movements (>2mm between successive scans); and interpolating missing middle scans by cubic spline. To achieve greater model generalizability, data augmentation was carried out, such as: coordinate systems rotations ([-15°C]); symmetric reflection of arch data; random noise addition (sigma=0.05mm); and synthetic data was made by biomechanical verified constraints.

## AI model architecture:

A hybrid deep learning model was designed that merged a spatial and temporal processing ability:

[1] Spatial feature extraction module: A three dimensional convolutional neural network that used the Point Net++ architecture processed a single tooth geometry and spatial relation in a dental arch. The input features were tooth surface meshes (2048 points per tooth), relative positions of neighboring teeth and occlusal relationships.

[2] Patient characteristic encoder: It is an encoder based on a multi-layer perceptron that utilizes patient-level characteristics such as demographics, malocclusion features and periodontal parameters to train an embedding used to generate a patient-specific embedding vector.

[3] Temporal sequence module: This is a bidirectional Long Short-Term Memory (LSTM) network that processed sequence movement data and identified temporal patterns in treatment response and allowed the model to learn with cumulative movement history.

[4] Movement prediction head: The movement prediction head was a fully connected network that used the contributions of all modules to produce six degrees of freedom predictions on the six degrees of freedom (mesio-distal, bucco-lingual, occluso-gingival and tip, torque, rotation) of each tooth.

[5] Attention mechanisms: Self-attention layers allowed the model to provide the relevance of each prediction to different teeth and stages of the treatment and the attention weightings offered interpretability to the clinical review of the model.

## Model training:

Patient-level stratified random sampling was applied to the dataset to obtain malocclusion type and appliance system proportional representation in the training (70), validation (15) and testing (15) sets. The training was done with Adam optimizer using initial learning rate of 5x 10 -4 and cosine annealing schedule. The loss function is comprised of mean squared error of continuous movement predictions and cross-entropy of movement direction classification. The maximum epochs were 200 and early stopping was done on validation loss (patience=20 epochs). The gradient accumulation of batch size was 64 cases as memory efficiency. A group of 4 NVIDIA A100 GPUs was trained on the PyTorch 2.0 with distributed data parallel processing. The time of total training was around 72 hours.

## Comparison methods:

The model predictions of AI were assessed versus two traditional methods:

[1] Pre-planned movement baseline: The comparison of movement with the predictions of commercial treatment planning software on case set-up

[2] Biomechanical calculation method: Predictions of physics based on published force-movement relationships and average biological response coefficients based on the orthodontic literature.

## Outcome measures:

## Primary outcomes included:

## Direction Accuracy:

Percentage of correctly predicted movement direction written directions per degree of freedom.

## Mean Absolute Error (MAE):

Mean value of absolute differences between movements that are predicted and achieved.

## Root Mean Square error (RMSE):

Square root of mean squared prediction error.

## Correlation Coefficient:

Pearson correlation of predicted movements and achieved movements. The secondary outcomes comprised of movement specific accuracy analysis, malocclusion type stratified performance and appliance system comparisons.

## Statistical analysis:

The statistical procedures were done with Python (NumPy 1.24, SciPy 1.10, scikit-learn 1.2). Mean and standard deviation were used to describe continuous variables. The paired t-test was applied in model comparison when the error was normally distributed whereas the Wilcoxon signed-rank test was used when the error was not normally distributed. There were several comparisons, which were corrected by Bonferonni. The agreement of the movements achieved and predicted was measured using the Bland-Altman analysis. The significance level was fixed at p= 0.05.

## Results:

The final dataset comprised 4,218 completed orthodontic cases from 4,218 unique patients. Mean patient age was 28.4±12.7 years (range: 12-67 years), with 58.3% female patients. Mean treatment duration was 18.2±6.4 months with an average of 8.4±3.2 intraoral scans per case. Malocclusion distribution included Class I (52.8%), Class II (35.4%) and Class III (11.8%). Detailed dataset characteristics are presented in Table 1. The AI model demonstrated superior prediction performance compared to both conventional methods across all outcome measures. Overall direction accuracy was 91.3% for the AI model compared to 74.6% for planned movement baseline and 68.2% for biomechanical calculations (p<0.001 for both comparisons). Mean absolute error for linear movements was 0.24±0.18mm for the AI model versus 0.52±0.34mm for planned baseline and 0.61±0.42mm for biomechanical method (p<0.001). For angular movements, MAE was 1.87±1.24°C for AI versus 3.42±2.18°C for planned baseline and 4.15±2.87°C for biomechanical method (p<0.001). Correlation coefficients between predicted and achieved movements were significantly higher for the AI model (r=0.89) compared to planned baseline (r=0.67) and biomechanical method (r=0.58) (p<0.001). Comprehensive performance comparison is shown in Table 2.

Movement-specific analysis revealed varying prediction accuracy across different movement types. Intrusion and extrusion movements demonstrated highest accuracy (93.8%), followed by mesio-distal translation (92.4%), bucco-lingual movement (91.2%), tipping (90.1%), torque expression (88.7%) and rotation (86.4%). Subgroup analysis by malocclusion type showed consistent AI model performance: Class I (91.8% accuracy), Class II (90.9% accuracy) and Class III (90.5% accuracy) with no statistically significant differences (p=0.234). Similarly, appliance system comparison revealed comparable performance between clear aligner cases (90.8% accuracy) and fixed appliance cases (92.1% accuracy, p=0.089). Age-stratified analysis demonstrated slightly higher accuracy in adult patients (>18 years: 92.1%) compared to adolescents (12-18 years: 89.8%, p=0.024). Crowding severity influenced prediction accuracy, with mild crowding cases showing highest accuracy (93.2%) compared to moderate (91.1%) and severe crowding (88.7%, p<0.001). Movement-specific performance data are presented in Table 3. Attention weight analysis revealed that the model assigned highest importance to: previous movement history (mean attention weight 0.28), adjacent tooth positions (0.22), tooth-specific morphology (0.18) and patient age (0.14). The temporal attention module demonstrated learning of biological response patterns, with higher weights assigned to movements occurring 2-4 weeks prior to the prediction time point.

## Discussion:

The paper involves the creation and testing of a state-of-the-art artificial intelligence model to predict three-dimensional tooth movement during orthodontic therapy, which proves to be much more effective than traditional prediction tools. The obtained accuracy of 91.3 percent and average error of 0.24mm in linear motions are considerable advances in comparison with the current methods [25]. This can be explained by the fact that the AI model is able to pick up the complicated patterns of the real treatment results and not based on theoretical biomechanical assumptions. The conventional prediction techniques are based on the assumption of equal biological response to orthodontic forces but clinical data indicate that there is a high level of individual variation in treatment response [26]. The deep learning approach allows the incorporation of patient-specific variables that alter treatment response that effectively personalizes the predictions based on learned patterns of similar cases [27]. The result obtained that the rotation movements had the lowest prediction accuracy (86.4) is consistent with clinical observations and earlier studies having established rotation as the most difficult type of movement to attain predictably [28]. Rotational movements include complicated force systems on teeth with different root morphologies and the distribution of periodontal ligament stress under rotation is significantly different compared with the distribution of periodontal ligament stress under translational movements [29]. Nevertheless, this relative weakness did not stop the AI model which significantly outperformed traditional approaches to the rotation prediction. The increased precision in intrusion and extrusion movements (93.8 percent) can be due to the fact that the movements are more restricted in only one axis, including the fact that biological responses to vertical forces are relatively consistent [30]. Studies have determined that vertical motions of the teeth are more predictable compared to horizontal or rotational motions, which is probably because the stress distribution is homogenous in the periodontal ligament during pure vertical loading [31]. The overall lizability of the created AI system is based on the consistency of the model performance of the various types of malocclusion. It has been argued in the literature that prediction accuracy can depend on the malocclusion severity and complexity, but this is not the case due to the high and diverse learning rates of the large and heterogeneous training dataset [32].

The clinical implications of this finding are significant because it implies that it is a reliable model when used irrespective of initial malocclusion classification. The minor inaccuracy of the adolescent patients relative to their adult counterparts could be related to the higher biological variability with regard to the growth related changes during treatment [33]. Active development has the potential to change the response to tooth movements by difference in rates of bone metabolism and continued development of the jaw. The use of growth prediction models or growth-specific characteristics can also enhance the accuracy in younger patients [34]. The severity of the crowding issue on the prediction accuracy points to the biomechanical intricacies of the problem of severe space discrepancies. In the severely crowded arches, teeth are subject to complicated force interactions with their neighboring teeth and the movement vectors cannot follow the intended direction because of these interproximal contacts [35]. To some extent, the ability of the AI model to include the location of neighboring teeth with the help of attention mechanisms is a solution to this problem, but additional optimization can be advantageous. The attention analysis is very insightful on the decision-making process of the model, which supports clinical interpretability. The great value given to past movement history is in line with the biological fact that teeth with positive initial response would respond well and those that are resistant would continue to be difficult during the treatment process [36]. This time-based learning allows the model to adjust the predictions according to the observed development of treatment. The similarity in the performance of clear aligner and fixed appliance cases, even though based on a different underlying biomechanics and force systems, indicates that the AI model has learned and applied the movements in a generalizational way across the type of appliances [37].

The implication of this finding is that it is possible to have a single prediction system that could be used across various treatment modalities which can be easily implemented in the clinic. The consequences of better prediction accuracy in the clinic are enormous. Fewer prediction errors mean fewer corrections made during the process, less refinement aligners are required, shorter care periods and patient contentment [38]. More precise predictions also allow making the discussion of informed consent better informed, since the clinicians will be able to give their patients realistic expectations about the outcomes of treatment and the timing [39]. The model architecture with attention mechanisms can be clinically adopted with benefits of offering interpretable to the user. Analysis of the weights of attention would help clinicians to determine what factors had the greatest impact on particular prediction, allowing them to make clinical judgment and identify instances in which predictions are less accurate [40]. Such openness will resolve the issues of black box AI systems used in the healthcare field. There are a number of limitations which should be considered. The retrospective design is also characterized by the possibility of selection bias since only the successfully completed cases were used. Terminated cases with poor response might possess systematically different factors that might influence model generalizability [41]. Also, the dataset included mostly North American and European patients and further external validation with less homogenous populations should be provided. The model needs digital records of treatment such as sequential intra oral scans, which can be restrictive in practices lacking digital workflow infrastructure. Moreover, real-time connecting with treatment planning tools and clinical decision support tools need further development [42]. The future research needs to be conducted on prospective validation, integration into commercial treatment planning systems and the extension of training data to cover different populations and treatment strategies [43].

## Conclusion:

We show the developed and validated an AI-based three-dimensional tooth movement prediction model that demonstrated high directional accuracy and low error for both linear and angular movements during orthodontic treatment. The model consistently outperformed conventional biomechanical calculations and commercial planning software across different types of tooth movements, malocclusions and appliance systems, with the highest accuracy observed for intrusion/extrusion movements. Thus, we show the clinical potential of AI-driven prediction systems to enhance orthodontic treatment planning accuracy, reduce treatment duration and improve overall patient outcomes.

## Figures and Tables

**Table 1:** Dataset demographics and clinical characteristics

**Characteristic**	**Total (n=4,218)**	**Clear Aligner (n=2,847)**	**Fixed Appliance (n=1,371)**	**p-value**
**Demographics**				
Age, mean±SD (years)	28.4±12.7	31.2±11.4	22.6±13.1	<0.001*
Female, n (%)	2,459 (58.3%)	1,724 (60.6%)	735 (53.6%)	<0.001*
**Malocclusion Type**				
Class I, n (%)	2,228 (52.8%)	1,567 (55.0%)	661 (48.2%)	0.002*
Class II, n (%)	1,494 (35.4%)	978 (34.4%)	516 (37.6%)	
Class III, n (%)	496 (11.8%)	302 (10.6%)	194 (14.2%)	
**Treatment Parameters**				
Treatment duration, mean±SD (months)	18.2±6.4	16.8±5.2	21.1±7.8	<0.001*
Number of scans, mean±SD	8.4±3.2	9.1±2.8	6.9±3.5	<0.001*
Teeth per case, mean±SD	26.8±1.4	26.9±1.3	26.6±1.6	0.042*
**Crowding Severity**				
Mild (<3mm), n (%)	1,245 (29.5%)	892 (31.3%)	353 (25.7%)	<0.001*
Moderate (3-6mm), n (%)	1,856 (44.0%)	1,312 (46.1%)	544 (39.7%)	
Severe (>6mm), n (%)	1,117 (26.5%)	643 (22.6%)	474 (34.6%)	
Initial Overjet (mm)	4.2±2.1	3.9±1.8	4.8±2.5	<0.001*
Initial Overbite (mm)	3.1±1.9	2.9±1.7	3.6±2.2	<0.001*
Statistically significant (p<0.05)

**Table 2:** Prediction performance comparison among methods

**Parameter**	**AI Model**	**Planned Baseline**	**Biomechanical Method**	**p-value (AI vs. Planned)**	**p-value (AI vs. Biomech)**
**Overall Accuracy**					
Direction accuracy (%)	91.3±4.2	74.6±8.5	68.2±9.8	<0.001*	<0.001*
**Linear Movements**					
MAE, mesio-distal (mm)	0.21±0.15	0.48±0.31	0.56±0.38	<0.001*	<0.001*
MAE, bucco-lingual (mm)	0.26±0.19	0.54±0.35	0.64±0.44	<0.001*	<0.001*
MAE, vertical (mm)	0.24±0.17	0.53±0.34	0.62±0.41	<0.001*	<0.001*
Overall linear MAE (mm)	0.24±0.18	0.52±0.34	0.61±0.42	<0.001*	<0.001*
RMSE linear (mm)	0.32±0.21	0.68±0.42	0.79±0.51	<0.001*	<0.001*
**Angular Movements**					
MAE, tip (°C)	1.72±1.18	3.24±2.05	3.98±2.74	<0.001*	<0.001*
MAE, torque (°C)	1.89±1.28	3.51±2.24	4.25±2.92	<0.001*	<0.001*
MAE, rotation (°C)	2.01±1.36	3.52±2.28	4.21±2.89	<0.001*	<0.001*
Overall angular MAE (°C)	1.87±1.24	3.42±2.18	4.15±2.87	<0.001*	<0.001*
RMSE angular (°C)	2.45±1.58	4.38±2.76	5.24±3.42	<0.001*	<0.001*
**Correlation (r)**					
Linear movements	0.91	0.69	0.61	<0.001*	<0.001*
Angular movements	0.87	0.65	0.55	<0.001*	<0.001*
Overall	0.89	0.67	0.58	<0.001*	<0.001*
MAE: Mean Absolute Error;
RMSE: Root Mean Square Error;
Statistically significant (p<0.05)

**Table 3:** Movement-specific prediction accuracy and subgroup analysis

**Category**	**Direction Accuracy (%)**	**Linear MAE (mm)**	**Angular MAE (°C)**	**p-value**
**Movement Type**				<0.001*
Intrusion/Extrusion	93.8±3.8	0.19±0.14	-	
Mesio-distal translation	92.4±4.1	0.22±0.16	-	
Bucco-lingual movement	91.2±4.5	0.27±0.19	-	
Tipping	90.1±4.8	-	1.68±1.12	
Torque expression	88.7±5.2	-	1.95±1.31	
Rotation	86.4±5.8	-	2.18±1.45	
Malocclusion Type				0.234
Class I	91.8±4.1	0.23±0.17	1.84±1.22	
Class II	90.9±4.3	0.25±0.18	1.89±1.25	
Class III	90.5±4.5	0.26±0.19	1.92±1.28	
Appliance System				0.089
Clear aligners	90.8±4.4	0.25±0.18	1.91±1.27	
Fixed appliances	92.1±4.0	0.22±0.17	1.82±1.21	
Patient Age				0.024*
Adolescent (12-18 years)	89.8±4.8	0.27±0.20	1.98±1.32	
Adult (>18 years)	92.1±3.9	0.22±0.16	1.79±1.18	
Crowding Severity				<0.001*
Mild (<3mm)	93.2±3.6	0.20±0.15	1.72±1.14	
Moderate (3-6mm)	91.1±4.2	0.24±0.18	1.86±1.24	
Severe (>6mm)	88.7±5.1	0.29±0.21	2.05±1.36	
Tooth Type				<0.001*
Incisors	92.4±3.9	0.22±0.16	1.78±1.18	
Canines	90.8±4.3	0.25±0.18	1.89±1.26	
Premolars	91.5±4.1	0.24±0.17	1.85±1.23	
Molars	89.2±4.8	0.28±0.20	1.98±1.32	
*MAE: Mean Absolute Error;
statistically significant (p<0.05)

## References

[1] Patil CD (2025). Bioinformation..

[2] Kavousinejad S (2024). Cureus..

[3] Nordblom NF (2024). J Dent Res..

[4] Ryu J (2023). Sci Rep..

[5] Van Nistelrooij N (2024). J Dent..

[6] Lahoud P (2021). J Endod..

[7] Baldini B (2025). J Dent..

[8] Wang X (2024). BMC Oral Health..

[9] Mengi A (2024). J Oral Biol Craniofac Res..

[10] Kapoor S (2023). J Orthod..

[11] Lin L (2023). Am J Orthod Dentofacial Orthop..

[12] Ruiz DC (2025). J Dent..

[13] Gurgel M (2024). Orthod Craniofac Res..

[14] Mohammad-Rahimi H (2021). Am J Orthod Dentofacial Orthop..

[15] Najeeb M, Islam S (2025). BMC Oral Health..

[16] Lin J (2025). BMC Oral Health..

[17] Hirata Y (2024). J Echocardiogr..

[18] Guinot-Barona C (2024). J Esthet Restor Dent..

[19] Fontenele RC (2022). J Dent..

[20] Ren R (2025). BMC Oral Health..

[21] Chang Q (2024). Orthod Craniofac Res..

[22] Mahto RK (2022). BMC Oral Health..

[23] Kazimierczak W (2024). J Clin Med..

[24] Gupta S (2025). J World Fed Orthod..

[25] Fontenele RC, Jacobs R (2025). Int Endod J..

[26] Gracea RS (2025). J Dent..

[27] Chen HC (2021). JMIR Mhealth Uhealth..

[28] Santos-Junior AO (2025). Sci Rep..

[29] Liu J (2021). Adv Clin Exp Med..

[30] Yoon K (2024). J Dent..

[31] Li S (2022). Biomed Res Int..

[32] Pang YN (2025). J Dent Sci..

[33] Lee SC (2022). Prog Orthod..

[34] Alam MK (2024). Heliyon..

[35] Kazimierczak N (2024). J Clin Med..

[36] Bardideh E (2024). Am J Orthod Dentofacial Orthop..

[37] Peyrin-Biroulet L (2024). United European Gastroenterol J..

[38] Tanaka OM (2025). Dental Press J Orthod..

[39] Chen W (2024). Front Cardiovasc Med..

[40] Kühnisch J (2022). J Dent Res..

[41] Capurro N (2024). Br J Dermatol..

[42] Bastiaansen WAP (2025). J Med Internet Res..

[43] Yacoub B (2022). AJR Am J Roentgenol..

